# Structure–Property Relationships of *Cyperus papyrus* Fibers as Sustainable Reinforcements for Polymeric Composites

**DOI:** 10.3390/polym18182219

**Published:** 2026-09-11

**Authors:** Mateus Urbano do Nascimento, Felipe Gabriel Santos Araújo, Hemanuelly Ferreira Breda Lan Oliveira, Felipe Perissé Duarte Lopes, Henry Alonso Colorado Lopera, Michel Picanço Oliveira

**Affiliations:** 1 Department of Chemical Engineering, Federal University of Espírito Santo, Alegre 29500-000, ES, Brazil; urbanomateus28@gmail.com (M.U.d.N.); hemanuelly.oliveira@edu.ufes.br (H.F.B.L.O.); 2 Department of Forestry and Wood Sciences, Federal University of Espírito Santo, Jerônimo Monteiro 29550-000, ES, Brazil; felipe.gabriel.araujo@gmail.com; 3 Materials Science and Engineering Department, State University of North Fluminense, Campos dos Goytacazes 28013-602, RJ, Brazil; perisse@uenf.br; 4 CCComposities Laboratory, University of Antioquia, Medellin 050010, Colombia; henry.colorado@udea.edu.co

**Keywords:** lignocellulosic fibers, long-fibers, mechanical characterization, chemical composition

## Abstract

The development of sustainable structural materials has driven interest in renewable plant fibers as alternatives to synthetic reinforcements. In this study, the structural, thermal, mechanical, and chemical characteristics of *Cyperus papyrus* fibers were comprehensively evaluated to assess their potential as reinforcement materials for polymeric composites. The fibers exhibited an average diameter of 164.87 µm, with tensile strength and Young’s modulus values of 141.95 ± 89.33 MPa and 11.15 GPa, respectively. Scanning electron microscopy revealed a rough and irregular surface morphology characteristic of untreated lignocellulosic fibers. X-ray diffraction analysis indicated a crystallinity index of 70.15%, while thermogravimetric analysis demonstrated stability up to 220 °C. Chemical composition analysis revealed high α-cellulose content (63.3%) and moderate lignin levels, contributing to a balance between stiffness and processability. The significant relationship observed between fiber diameter and tensile behavior, together with the structural, chemical, and thermal characteristics of the fibers, provides a broader understanding of the structure–property relationships relevant to their potential use as sustainable reinforcement materials. Overall, *C. papyrus* fibers represent a promising bio-derived material for environmentally conscious engineering applications.

## 1. Introduction

The use of natural fibers as reinforcing elements in composites has attracted growing attention in recent years due to the environmental, economic, and performance benefits these materials can offer [1,2]. In the context of advanced materials research, natural fibers have emerged as sustainable alternatives to conventional synthetic reinforcements, such as glass, carbon, and petroleum-derived polymers. This approach supports the development of high-performance and eco-efficient structural materials through a deeper understanding of their physical, chemical, and mechanical characteristics. Therefore, the exploration of lignocellulosic materials as natural reinforcements is essential for technological progress in composite science and engineering [3,4,5].

Plant-based fibers such as sisal, jute, hemp, flax, and bamboo have been extensively characterized for their structural, mechanical, and thermal properties, each exhibiting distinct features that make them suitable for specific applications [6,7,8] in sectors including automotive, construction, aerospace, sports and leisure, packaging, and furniture industries. They are also employed in coatings, insulation materials, structural components, and consumer products [8], highlighting their versatility and potential in the development of advanced bio-based composite systems [9,10].

As highlighted by Sheferaw et al. [11], growing environmental concerns associated with synthetic fibers, together with the increasing production costs of conventional cellulosic fibers, reinforce the need to explore new sustainable and eco-friendly sources of natural reinforcements. Among the emerging lignocellulosic resources, *Cyperus papyrus* has attracted increasing attention because of its rapid growth, abundance in wetlands, and suitability as a low-cost renewable fiber source [12].

Historically, papyrus has been used in the manufacture of paper, boats, mats, and baskets, whereas more recent studies have demonstrated its potential for textile applications [11], poly(lactic acid) composites [13], and natural rubber composites for shoe soles [12]. Furthermore, papyrus fibers exhibit mechanical characteristics comparable to those of conventional reinforcement fibers such as jute, sisal, and hemp [12], while their culms consist of long fibers composed primarily of cellulose and lignin [14]. Taken together, these characteristics indicate that *C. papyrus* is a promising renewable resource for the development of sustainable polymeric composites.

Despite these promising characteristics, the available literature on *C. papyrus* remains fragmented, with previous studies focusing mainly on specific applications or individual fiber properties. A comprehensive assessment integrating fiber morphology, chemical composition, thermal behavior, cellulose structural organization, and mechanical performance is still limited. In particular, the influence of the natural variation in fiber diameter on tensile behavior has not been sufficiently addressed for *C. papyrus*. Establishing these relationships is important because the performance of lignocellulosic fibers as potential reinforcements depends not only on their cellulose content, but also on the combined effects of their structural, physical, thermal, and mechanical characteristics.

Accordingly, the present study aims to investigate the mechanical, thermal, physical, and chemical properties of papyrus fibers, as well as to examine the correlation between fiber diameter and mechanical performance. The findings provide a comprehensive understanding of the structure–property relationships of papyrus fibers and support their evaluation as sustainable reinforcements for polymeric composites.

## 2. Materials and Methods

### 2.1. Fiber Extraction

Papyrus was collected in the municipality of Venda Nova, Espírito Santo, Brazil (−20.411317, −41.109097), from an agro-industrial effluent treatment pond. The fibers were obtained from the plant stem, which, due to its long length, was cut into sections of approximately 50 cm to facilitate transport and storage.

For plant defibration, the epidermis of the papyrus stems was removed, exposing the fibers covered by mucilage, which was then mechanically stripped using a plastic-bristle brush. The fibers were subsequently washed to remove mucilage residues, beaten to prevent entanglement, and air-dried until reaching equilibrium moisture content.

### 2.2. Fiber Diameter Variation

**Figure 1:**
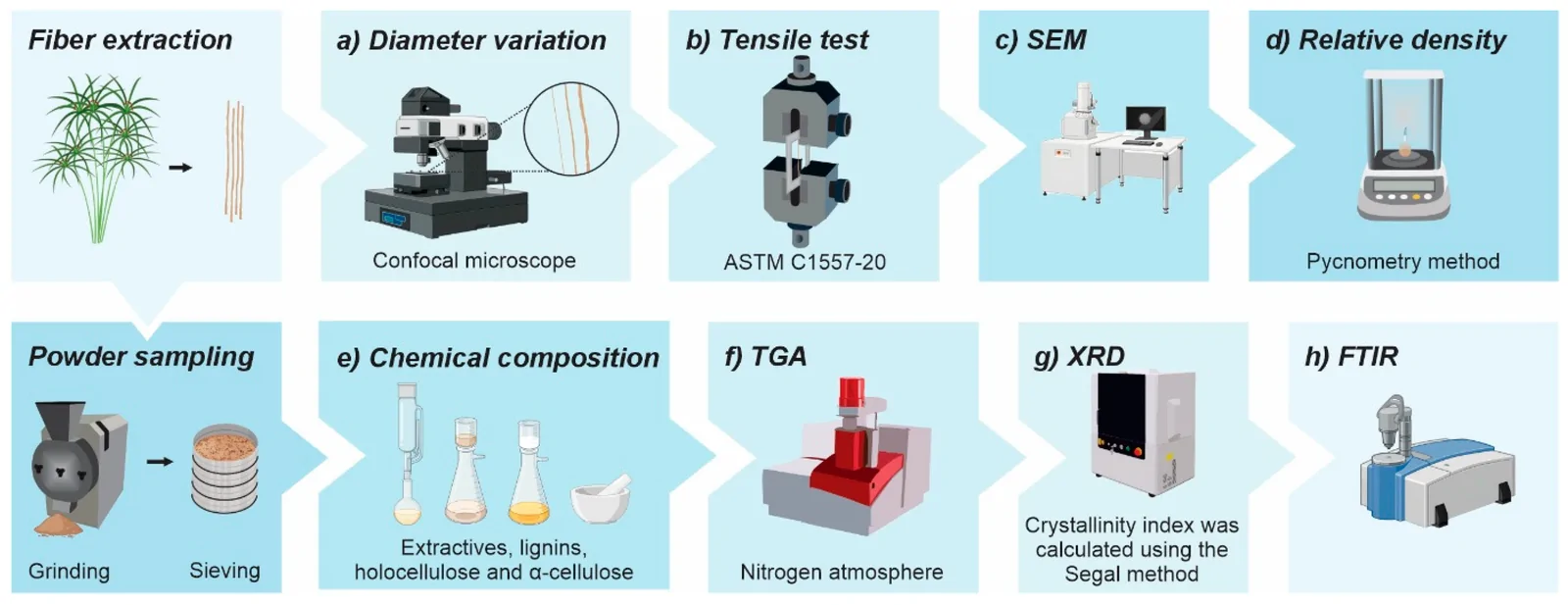
Scheme of papyrus fiber characterization: diameter variation (**a**), tensile strength (**b**), surface morphology by SEM (**c**), relative density (**d**), chemical characterization (**e**), thermogravimetric analysis (**f**), X-ray diffraction analysis (**g**), Fourier transform infrared spectroscopy (**h**).

The diameter of one hundred randomly selected fibers was measured (Figure 1b). The measurements were performed using an Olympus confocal microscope (Olympus, Nagano, Japan) with the equipment’s software tools at a 118× objective magnification. The results were plotted in a histogram of diameter variation according to statistical parameters, with Sturges’ method being used to determine the number of classes as it best represents the observed frequency distribution.

### 2.3. Tensile Test

The tensile mechanical properties (Figure 1b) of the fibers were evaluated using an Oswaldo Filizola universal testing machine (Oswaldo Filizola, São Paulo, Brazil), model AM2KN. A total of forty fibers were mechanically tested following the guidelines of ASTM C1557 [15]. The fibers were previously classified into eight diameter classes based on the frequency distribution obtained from the diameter measurements, with five fibers tested in each class. The fibers were mounted with a gauge length of 65 mm and tested at a crosshead speed of 1 mm min^−1^.

The normality of the residuals and homogeneity of variances were assessed using the Shapiro–Wilk and Levene tests, respectively. Differences in tensile strength among diameter classes were evaluated by one-way analysis of variance, followed by the Scott–Knott mean grouping test at the 5% significance level. In addition, the relationship between fiber diameter and tensile strength was assessed using Spearman’s rank correlation. All statistical analyses were performed using R software 5.0 [16].

### 2.4. Scanning Electron Microscopy (SEM)

Fiber samples were morphologically analyzed using scanning electron microscopy (Figure 1c) with secondary electrons on a JEOL JSMIT200 model (Tokyo, Japan). For analysis, the samples were first mounted on sample holders and coated with gold to ensure the material’s electrical conductivity.

### 2.5. Relative Density

The fiber density was determined using the pycnometry method (Figure 1d), employing 25 mL pycnometers, water as the fluid, and approximately 1.5 g of fiber. After measuring the mass of the pycnometers containing water and fibers, the pycnometer containing only water was weighed. Following the mass measurements, five independent measurements were performed, and the average density value was calculated according to Equation (1):

(1) $$\rho_{r}=\left[\frac{m1}{m2+m3-m4}\right]\times \rho_{a},$$

where $\rho_{r}$ is the true density; m1 is the mass of the dry sample; m2 is the mass of the fluid; m3 is the mass of the sample; m4 is the final mass of the sample and fluid; and $\rho_{a}$ is the density of water.

### 2.6. Chemical Composition

The extractive content was determined according to TAPPI 204 cm-97 [17], using sequential extractions with acetone and hot water, followed by oven drying and gravimetric determination. Insoluble lignin (Klason lignin) was quantified following TAPPI 222 om-02 [18], with acid hydrolysis using 72% sulfuric acid, while soluble lignin was measured by UV-Vis spectrophotometry at 205 nm; total lignin corresponded to the sum of both fractions. Holocellulose content was obtained by the Wise et al. [19] method based on acid delignification with sodium chlorite and acetic acid. Alpha-cellulose was determined according to TAPPI 203 cm-99 [20], and hemicellulose was calculated by the difference between holocellulose and alpha-cellulose contents. All chemical composition analyses were performed in triplicate.

### 2.7. Thermal Analysis

The thermal degradation behavior and thermal stability of the fibers were assessed using Thermogravimetric Analysis (TGA) and Derivative Thermogravimetry (DTG) (Figure 1f). The measurements were performed using a LabSys Evo Thermogravimetric Analyzer (LabSys, Caluire, France) in the temperature range of 23 °C to 800 °C with a heating rate of 10 °C min^−1^ under an inert nitrogen atmosphere.

### 2.8. X-Ray Diffraction (XRD)

X-ray diffraction patterns of *C. papyrus* fibers were obtained using a Rigaku Miniflex-600 diffractometer (Rigaku, Tokyo, Japan) equipped with a D/teX Ultra detector and operating in θ–2θ reflection geometry. The instrument used a fixed divergence slit and Cu Kα radiation (λ = 1.5418 Å), achieved by suppressing the Cu Kβ component through a nickel filter. Measurements were carried out from 5° to 70° (2θ) with a step size of 0.02° and a scan rate of 10° min^−1^, resulting in a total acquisition time of approximately 6.5 min. Samples were mounted on a glass sample holder. The crystallinity index (CI) of the fibers was calculated using the method proposed by Segal et al. [21], according to Equation (2):

(2) $$I_{c}=\left[\frac{I_{200}-\;I_{am}}{I_{200}}\right]\times 100,$$

where $I_{200}$ is the maximum intensity of the principal peak with crystallographic plane 200, which refers to the fraction of crystalline cellulose in the material; $I_{am}$ is the intensity at the minimum between the 110 and 200 peaks, about 18.5°, that represents amorphous cellulose.

### 2.9. Fourier Transformed Infrared (FTIR) Spectroscopy

Fourier transform infrared spectra (Figure 1h) were recorded using a Bruker Tensor 27 spectrophotometer (Billerica, MA, USA) at room temperature over a frequency range of 400–4000 cm^−1^, with a resolution of 4 cm^−1^. The samples were analyzed using the attenuated total reflectance (ATR) technique.

## 3. Results and Discussion

### 3.1. Fiber Diameter Distribution

**Figure 2:**
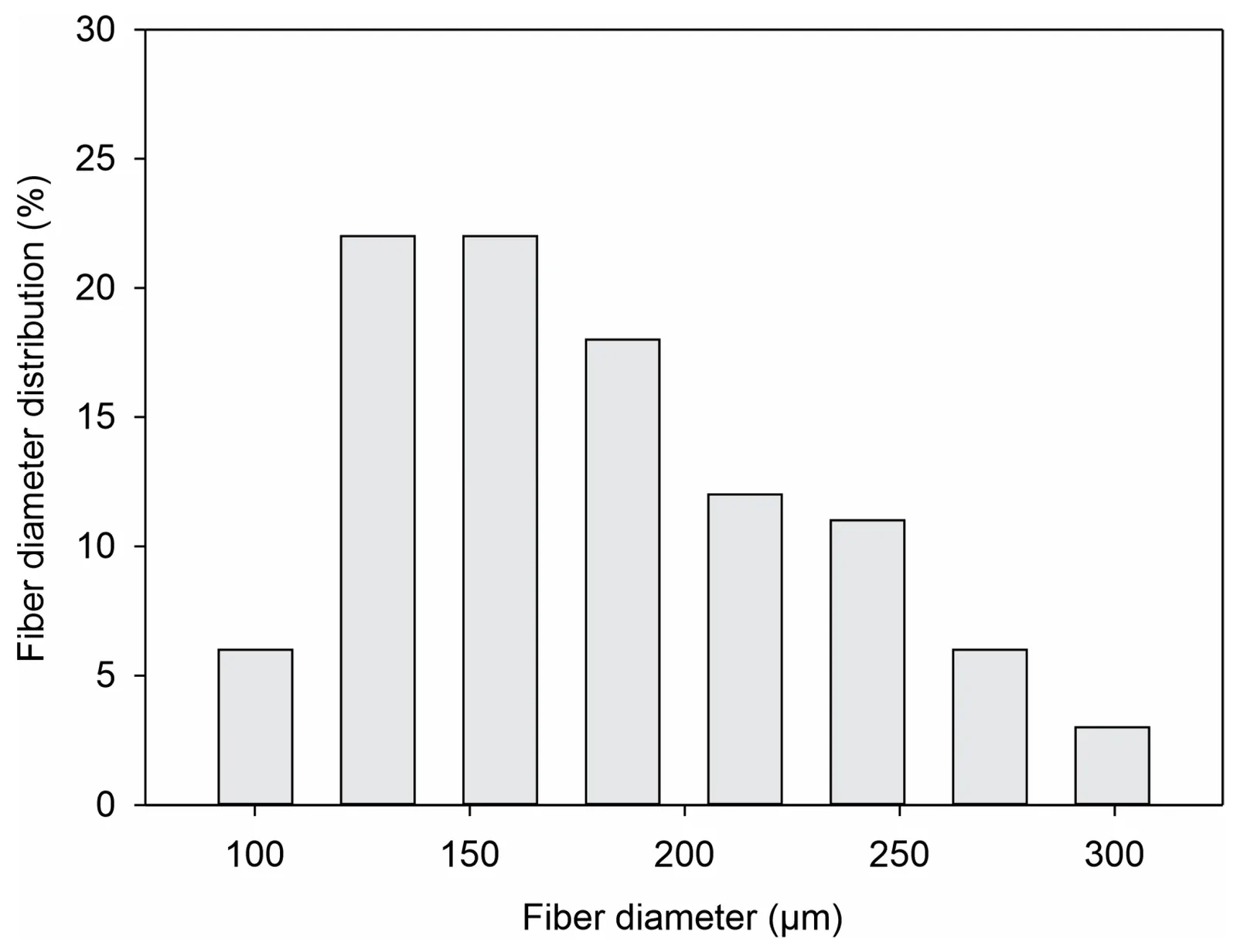
Diameter distribution frequency of papyrus fibers.

Fiber characterization regarding dimensions was performed based on measurements of 100 randomly selected fibers and organized in the histogram shown in Figure 2. The analyzed papyrus fibers exhibited an average diameter of 164.87 µm, with a range from 71.65 µm to 299.44 µm.

These dimensions differ from those reported by Sheferaw et al. [11], who evaluated the diameter of papyrus fibers extracted through the retting process, which consists of microbial degradation of the mucilage by immersing the plant in water for a specific period, and obtained an average result of 32.37 µm, significantly lower than those observed in the present work. However, the natural variation in fiber diameter must be considered, as the retting extraction process removes impurities and other surface constituents [22], consequently reducing thickness.

For composite applications, fiber diameter and aspect ratio are important geometrical parameters because they influence the available interfacial area and the efficiency of stress transfer between the matrix and the reinforcement. In general, finer fibers with a higher aspect ratio may provide more effective load transfer, although variability in fiber dimensions can also contribute to stress concentrations and premature failure [23]. Therefore, fiber diameter variation should be considered when evaluating lignocellulosic fibers for composite reinforcement.

Unlike synthetic fibers, which exhibit greater homogeneity in diameter, natural fibers exhibit substantial variability in their dimensions and mechanical properties as a consequence of their biological origin and the conditions associated with plant growth and fiber processing [24]. Factors such as plant maturity, growing conditions, extraction procedures, and morphological characteristics of the stem may therefore contribute to differences in fiber dimensions. Variability can also occur within the same plant according to the position of the fiber along the stem. Hue et al. [25], for example, evaluated flax fibers obtained from the top, middle, and bottom regions of the stem and observed differences in fiber dimensions and mechanical behavior, with the highest tensile strength and Young’s modulus generally occurring in fibers from the middle region. Stem morphology may represent an additional source of variation, since differences in stem diameter have also been associated with changes in fiber dimensions and mechanical properties [26]. These findings indicate that both sampling position and stem characteristics should be considered when interpreting the dimensional and mechanical variability in natural fibers.

To further investigate the influence of fiber diameter on the mechanical properties, the measured diameters were grouped into eight classes according to the Sturges criterion. The diameter intervals defining each class are presented in Table 1.

### 3.2. Mechanical Strength

**Figure 3:**
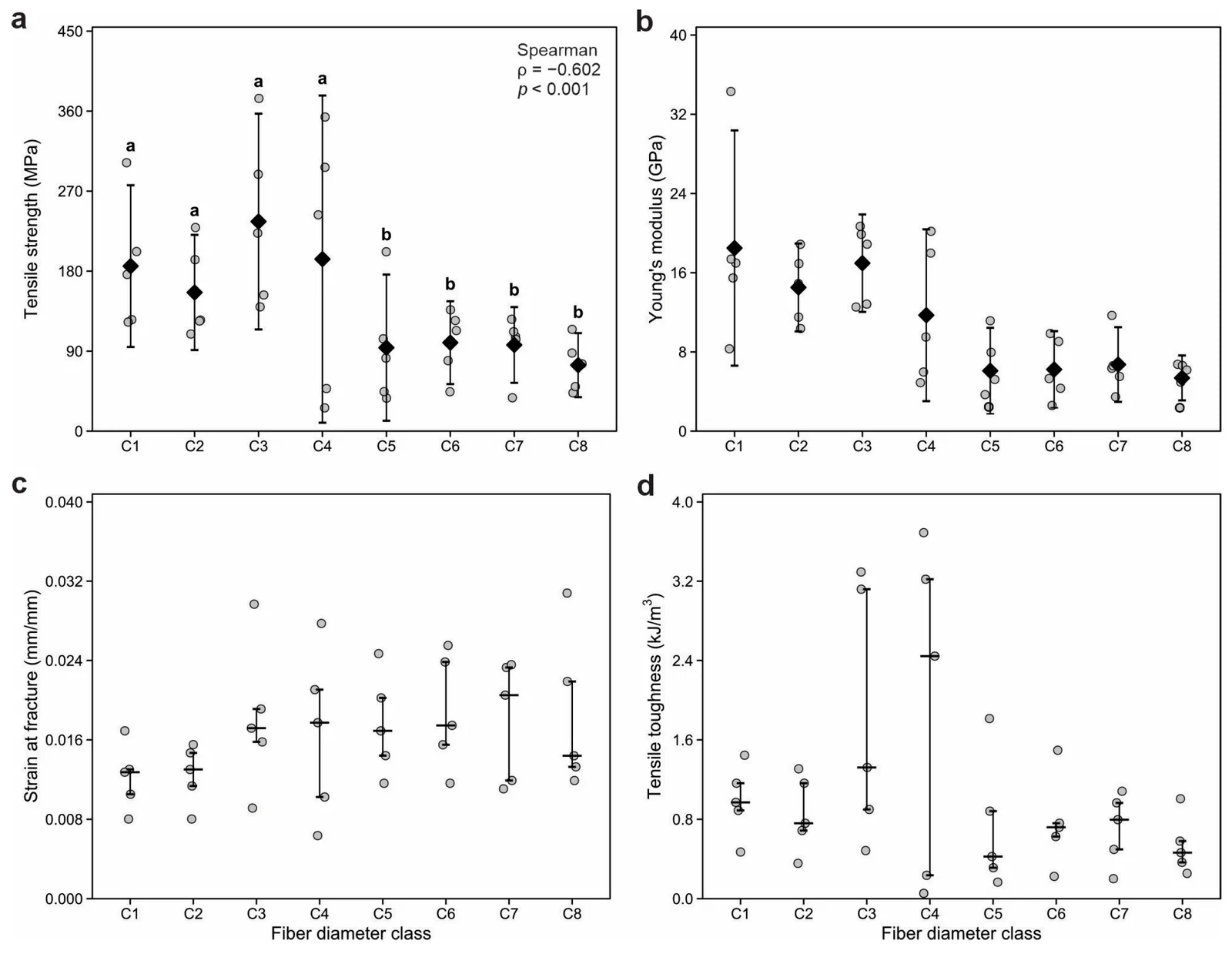
Mechanical properties of papyrus fibers as a function of diameter: tensile strength (**a**), Young’s modulus (**b**), strain at fracture (**c**), and tensile toughness (**d**). Circles represent individual fibers. Diamonds indicate class means with 95% confidence intervals. Horizontal bars represent medians and vertical bars represent the interquartile range. Different letters indicate Scott–Knott groups (*p* < 0.05).

The mechanical properties of the fibers grouped into the eight diameter classes defined according to the Sturges criterion (Table 1) are presented in Figure 3. The assumptions of normality and homogeneity of variances were satisfied according to the Shapiro–Wilk (*p* = 0.696) and Levene (*p* = 0.071) tests, respectively, supporting the application of ANOVA. The analysis revealed significant differences in tensile strength among the diameter classes (*p* = 0.0175). According to the Scott–Knott mean grouping test (5% significance level), the fibers were separated into two statistically distinct groups. The first group comprised classes C1–C4, corresponding to diameter intervals from 71.65 to 196.72 µm (Table 1), with mean tensile strengths between 155.98 and 235.79 MPa, whereas the second group included classes C5–C8, corresponding to diameter intervals greater than 196.72 µm and up to 321.79 µm, which exhibited significantly lower tensile strengths, ranging from 74.25 to 99.54 MPa.

The inverse relationship between fiber diameter and tensile strength was further confirmed by Spearman’s rank correlation (ρ = −0.602, *p* < 0.001), indicating a significant negative monotonic association between these variables. Fibers with smaller diameters generally exhibited superior tensile performance.

Similarly, the elastic modulus (Figure 3b) is higher for smaller-diameter fibers, reaching a maximum value of 18.48 ± 4.27 GPa. However, properties such as maximum strain (Figure 3c) and toughness (Figure 3d) do not show a correlation with diameter. Considering all fibers tested, the average tensile strength was 141.95 ± 89.33 MPa (coefficient of variation = 62.9%), while the average elastic modulus was 11.15 GPa.

The dispersion of the individual observations shown in Figure 3a reflects the intrinsic heterogeneity of papyrus fibers, which is characteristic of lignocellulosic materials. The relatively high coefficient of variation (62.9%) observed for tensile strength is attributed to the natural variability in anatomical features, including cell wall thickness, lumen dimensions, microfibril angle, and the occurrence of structural defects formed during plant growth. These characteristics are inherent to natural fibers and result in variations in load-bearing capacity even among fibers with similar diameters. Similar levels of variability have been reported for other natural fibers. For example, elementary flax fibers exhibited a coefficient of variation of 56.9%, which was associated with differences in cell wall composition, microfibril orientation, and microstructural defects [27]. Likewise, an interlaboratory study reported coefficients of variation ranging from 32 to 59% for flax fibers and from 51 to 80% for hemp fibers, highlighting that this level of variability is an inherent characteristic of individual lignocellulosic fibers rather than an experimental limitation [28].

Beyond the intrinsic variability associated with natural fibers, fiber diameter itself may influence tensile behavior. Larger-diameter fibers or fiber bundles present a higher probability of containing defects and structural irregularities that can act as failure-initiation sites, contributing to lower tensile strength [29]. From the perspective of composite reinforcement, smaller fiber diameters also provide a higher surface-area-to-volume ratio, increasing the available contact area with the matrix and potentially favoring interfacial bonding [30].

An inverse relationship between fiber diameter and tensile strength was also reported by Neuba et al. [31] for *Cyperus malaccensis* fibers. The authors observed statistically significant differences among diameter classes, with higher tensile strengths occurring in the smaller-diameter fibers. The significant negative correlation obtained in the present study is consistent with this behavior, indicating that diameter is an important source of mechanical variability in lignocellulosic fibers.

Regarding Young’s modulus, the present results indicate higher values in the smaller-diameter fiber classes. A similar tendency was reported by Hue et al. [25] for elementary flax fibers, in which smaller fiber diameters were associated with higher Young’s modulus and a lower occurrence of structural defects. Stochioiu et al. [32] also identified fiber diameter as an important factor influencing the stiffness of technical flax and hemp fibers; however, the high variability observed in their results indicated that mechanical behavior cannot be attributed to fiber diameter alone. Therefore, the relationship between diameter and stiffness should be interpreted together with other structural and morphological sources of variability.

### 3.3. SEM

**Figure 4:**
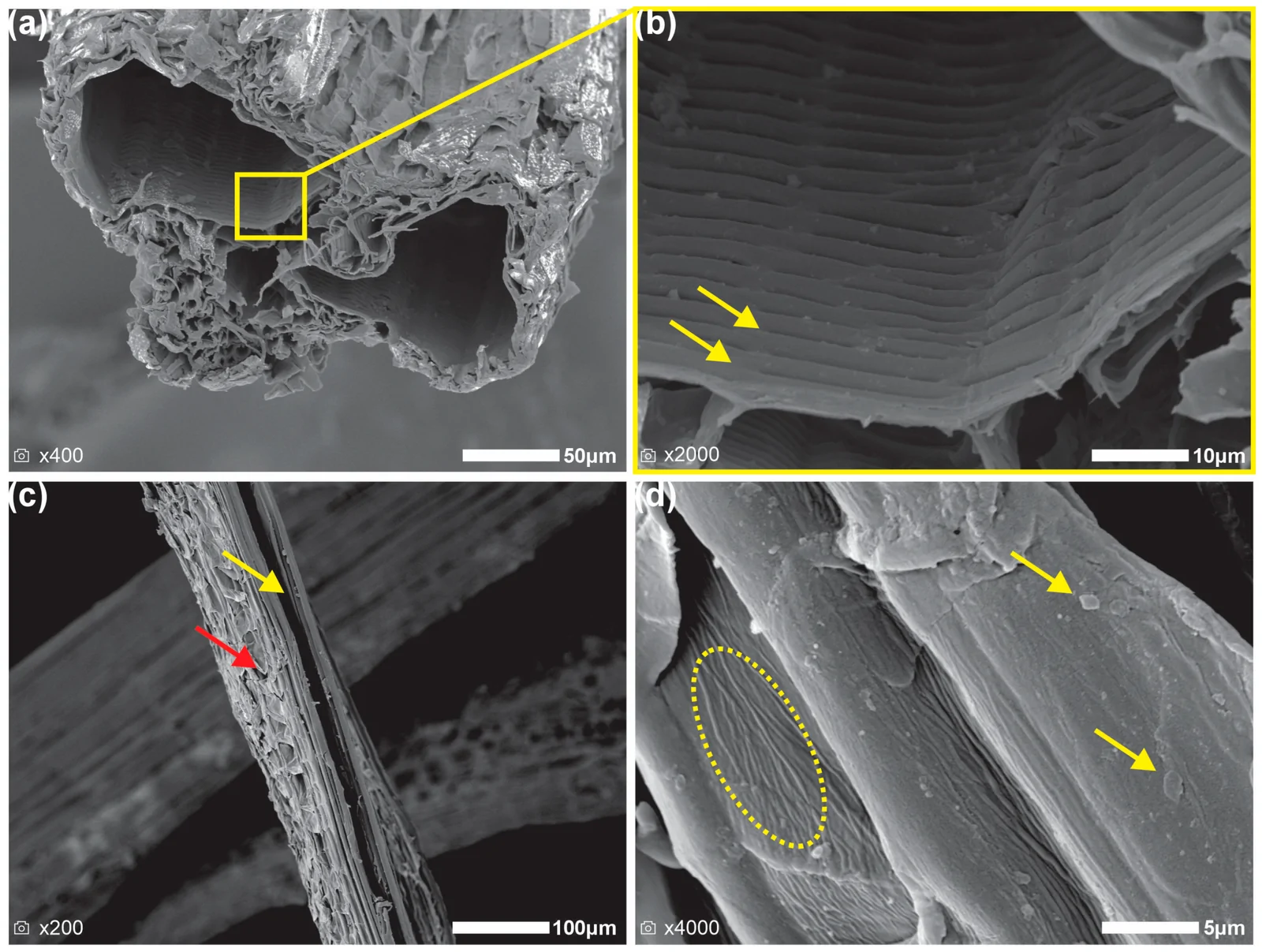
SEM micrographs of *Cyperus papyrus* fibers: (**a**) transverse section showing the heterogeneous internal morphology and large cavities; (**b**) detail of the inner surface of an internal cavity showing a scalariform-like pattern (yellow arrows); (**c**) longitudinal fiber surface showing distributed surface roughness (red arrow) and a localized surface defect (yellow arrow); and (**d**) higher-magnification view showing longitudinally oriented surface structures (dotted oval) and surface-adhered residues (yellow arrows).

Figure 4 shows the morphological characteristics of *C. papyrus* fibers at different observation scales. The transverse section reveals a heterogeneous and porous internal morphology, characterized by large cavities of irregular geometry separated by wall-like structures (Figure 4a). At higher magnification, the inner surface of one of these cavities exhibits regularly arranged transverse structures, forming a scalariform-like pattern (Figure 4b). Similar internal morphological features have been described in anatomical studies of *Cyperus* species [33].

The longitudinal surface of the fiber exhibits a heterogeneous morphology with pronounced irregularities (Figure 4c). Longitudinal grooves and ridges distributed along the surface contribute to its characteristic roughness. In contrast to this general surface roughness, localized discontinuities can also be observed, such as the feature indicated by the yellow arrow in Figure 4c, which was classified as a surface defect. Although such localized defects may be associated with the intrinsic fiber structure or may arise from mechanical damage during extraction and handling, their origin cannot be conclusively determined from the SEM observations alone. Similar surface deformations have been reported for other lignocellulosic fibers [34].

At higher magnification (Figure 4d), the fiber exhibits longitudinally oriented surface structures and small amounts of material adhered to the surface. Because the chemical nature of this material is not specifically determined, it is described here as surface-adhered residues rather than assigned to a particular fiber constituent. The heterogeneous and rough surface morphology observed in Figure 4c, d may be relevant to the use of these fibers as reinforcement in polymer composites. Surface roughness can increase the available contact area and favor mechanical interlocking between the fiber and polymer matrix [35], whereas surface-adhered residues may interfere with direct fiber–matrix contact and potentially affect interfacial adhesion [36,37]. However, these effects were not experimentally evaluated in the present study.

### 3.4. Chemical Composition and Relative Density

The main parameters of the chemical composition and density of fibers from species of the genus *Cyperus* are presented in Table 2. In the present study, *C. papyrus* exhibited an α-cellulose content of 63.3%, a value higher than that reported by Sheferaw et al. [11] for the same species and considerably greater than that described for *C. esculentus* by Pelegrin et al. [38]. This result is particularly relevant because α-cellulose is the fraction of greatest interest for the production of cellulosic derivatives, including nanocrystals and nanofibrils, and contributes to the mechanical strength and dimensional stability of lignocellulosic materials. The versatility of cellulose also extends to polymeric composites, where cellulose-based structures have been explored as functional components for tailoring properties such as thermal transport and flame-retardant performance [39,40].

Regarding hemicellulose, the present study reported 25.9% for *C. papyrus*, a value substantially higher than that found by Sheferaw et al. [11], and also above that recorded for *C. esculentus* and *C. dichrostachus* (Table 2). The high hemicellulose fraction may enhance fiber flexibility, although it may also result in lower thermal resistance, since this constituent is more susceptible to degradation. In contrast, the lignin content was lower than that described by Sheferaw et al. [11] and by Pelegrin et al. [38], but close to that reported by Baye and Tesfaye [41] for *C. papyrus*, *C. esculentus*, and *C. dichrostachus*, respectively. These results indicate a relatively moderate lignin content in *C. papyrus* compared with the other *Cyperus* fibers evaluated in Table 2.

With respect to extractives, the present study reported the highest value among the species, whereas Sheferaw et al. [11] and Baye and Tesfaye [41] reported considerably lower values of 2.75% and 1.55%, respectively. This higher number of extractives may influence compatibility with polymeric matrices, acting both as a chemical barrier and as a natural plasticizer. Although high extractive contents may compromise interfacial adhesion, their presence is also associated with bioactive compounds of industrial interest.

The values obtained for relative density are close to those described in the literature for lignocellulosic fibers in general [42,43,44]. The present study found 1.12 g/cm^3^ for *C. papyrus*, a result similar to that reported by Sheferaw et al. [11] and close to that recorded by Baye and Tesfaye [41] for *C. papyrus* and *C. dichrostachus*, respectively. This density range suggests that the evaluated fibers present characteristics compatible with their use as reinforcement in lightweight biocomposites intended for structural or semi-structural applications.

The differences observed among the studies presented in Table 2 should be interpreted considering the intrinsic variability of lignocellulosic materials. The chemical composition of natural fibers is strongly influenced by several factors, including plant maturity, genetic variability, geographical origin, edaphoclimatic conditions, harvest season, and the analytical methodology employed. Consequently, differences in the proportions of α-cellulose, hemicellulose, lignin, extractives, and even relative density are expected among studies, even when the same species is evaluated. Therefore, the higher hemicellulose and extractive content observed in the present study, together with the differences found for the other chemical constituents, should be interpreted within the context of this natural and methodological variability. Nevertheless, the high α-cellulose content, combined with the adequate relative density, reinforces the potential of *C. papyrus* fibers as a renewable raw material for paper, cellulose derivatives, polymer composites, and high-value lignocellulosic nanomaterials.

### 3.5. Thermal Analyses

**Figure 5:**
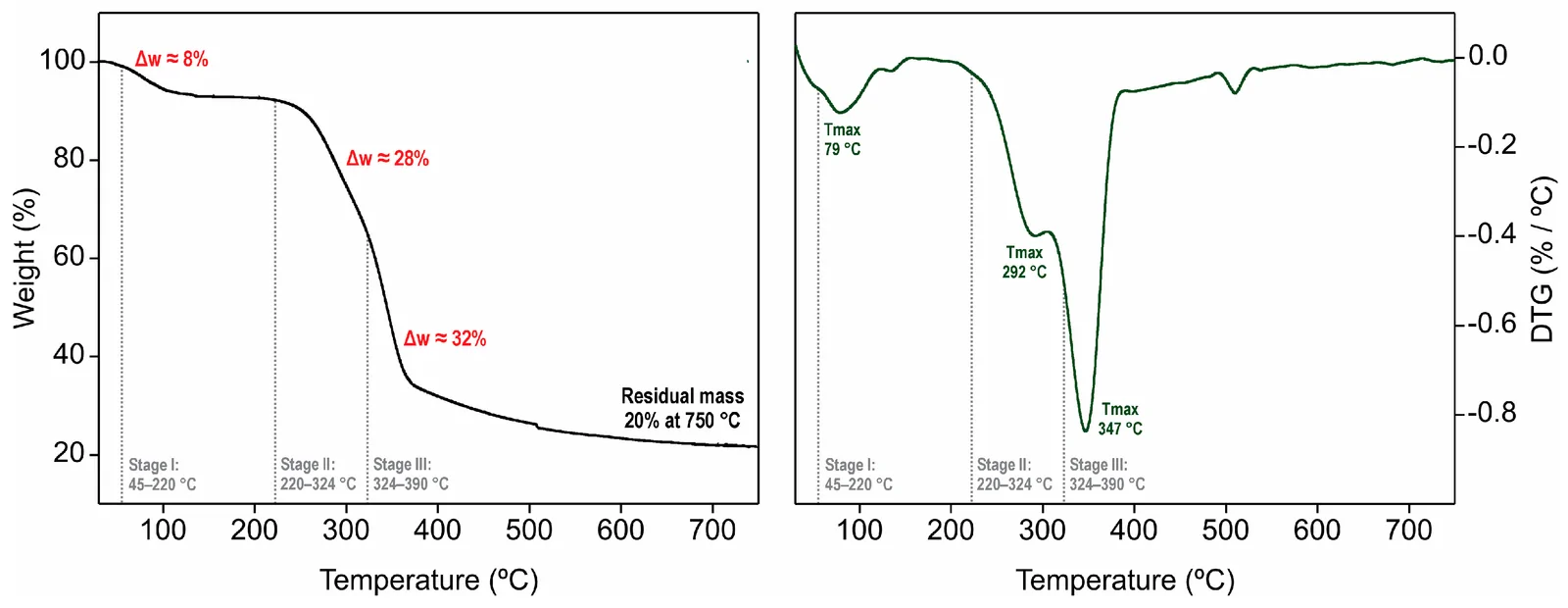
TGA and DTG curves of papyrus fibers.

TGA was performed to investigate the thermal stability of papyrus fibers. The thermal performance of the fiber is essential to determine its suitability for high-temperature applications. The thermogram (TGA) and derivative thermogravimetry (DTG) are shown in Figure 5.

From Figure 5, it can be observed that papyrus fiber degradation occurred in three stages. The first stage showed a mass loss of approximately 8% from 45 °C to 220 °C, with a maximum mass-loss rate (Tmax) at approximately 79 °C, as indicated by the DTG curve, which is related to the evaporation of water and volatile extractives. At this stage, no significant degradation of the lignocellulosic framework occurs, indicating that the structural components of the fiber remain essentially preserved. Above the moisture loss temperature, thermal stability gradually decreases, marking the onset of the main thermal degradation process, which occurs between 220 °C and 324 °C, with a 28% mass loss and a maximum mass-loss rate (Tmax) at approximately 292 °C. This stage is predominantly associated with hemicellulose degradation [45]. Due to its predominantly amorphous structure and lower thermal stability, hemicellulose undergoes depolymerization and cleavage of glycosidic linkages more readily than cellulose, resulting in the release of volatile products such as carbon dioxide, carbon monoxide, acetic acid, and other oxygenated compounds [46]. This behavior explains the pronounced mass loss observed in this temperature range and is consistent with the thermal degradation mechanisms reported for lignocellulosic biomass.

The next stage occurred between 324 °C and 390 °C, with a mass loss of approximately 32%. The DTG curve showed the most pronounced degradation peak, with a maximum mass-loss rate (Tmax) at approximately 347 °C. This stage is predominantly associated with cellulose degradation, which occurs through cleavage of β-1,4-glycosidic bonds followed by depolymerization of the polymer chains, generating volatile products and carbonaceous residues (char) [46,47]. The higher thermal resistance of cellulose is attributed to its semicrystalline structure, whose crystalline domains hinder heat transfer and delay thermal decomposition compared with hemicellulose [48]. Lignin degradation overlaps the cellulose-dominated thermal region because lignin decomposes slowly over a considerably broader temperature range than cellulose and hemicellulose [49]. In addition, interactions between lignin and cellulose can modify their pyrolytic behavior and promote secondary decomposition reactions [50].

After 400 °C, the mass loss rate decreased, indicating that most of the readily degradable lignocellulosic components had already decomposed. At 750 °C, approximately 20% of the initial mass remained as thermal residue. The remaining mass is predominantly associated with carbonaceous residues and slowly degrading lignin-derived structures. Lignin undergoes gradual thermal decomposition over a considerably broader temperature range than cellulose, extending well beyond the main cellulose degradation region [51]. This behavior is consistent with the gradual residual mass loss observed at higher temperatures. Thermal stability of lignocellulosic fibers is a challenge for their use in composites, as natural fibers are prone to decomposition at elevated temperatures during composite manufacturing, such as during thermoset polymer curing or thermoplastic extrusion, which impacts fiber characteristics and matrix interaction [52]. Thus, based on the onset of the main thermal degradation stage, papyrus fibers can be considered thermally stable up to approximately 220 °C. Similar thermal stability was found by Mayandi et al. [53] for *C. pangorei* fibers.

### 3.6. X-Ray Diffraction

**Figure 6:**
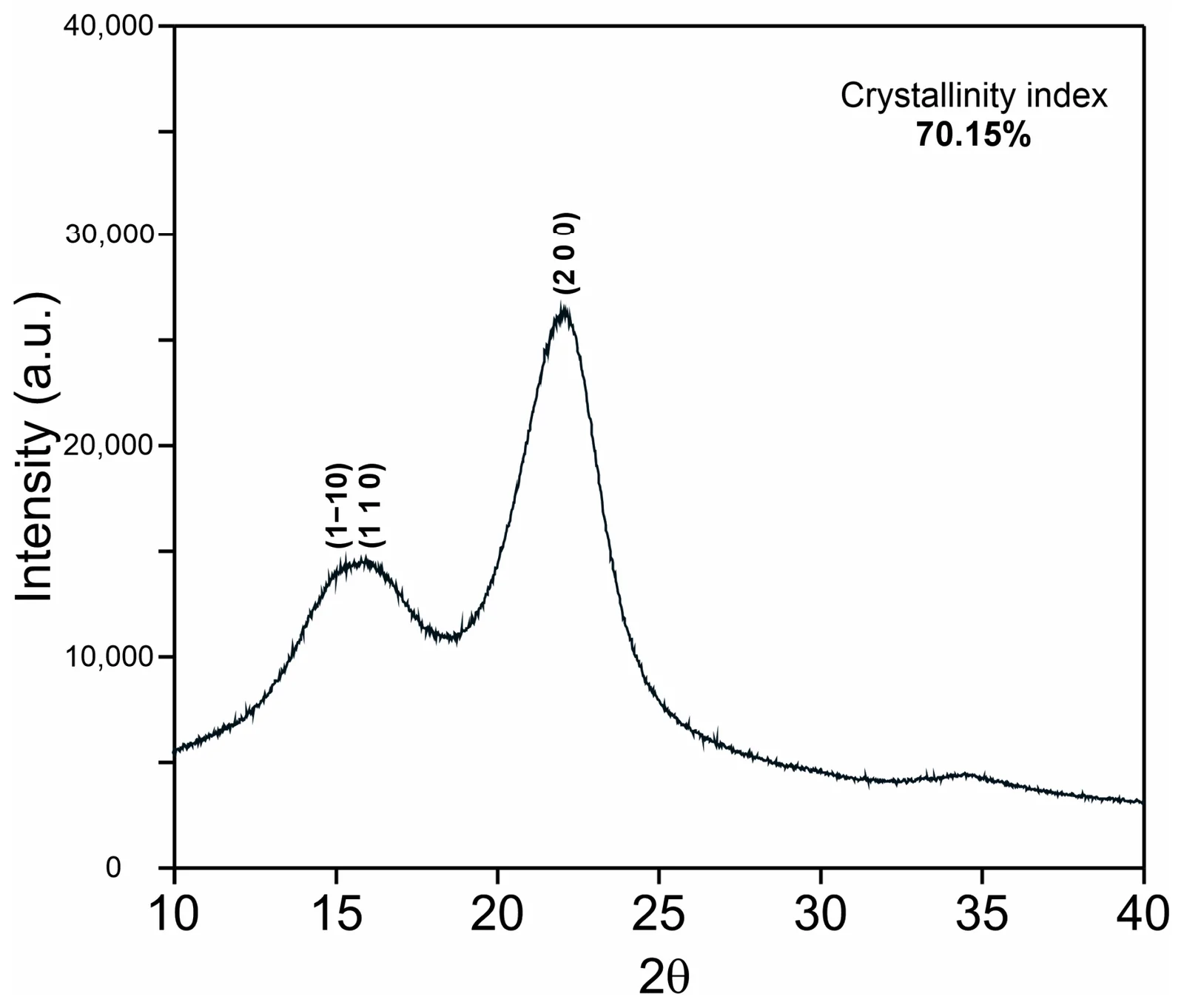
X-ray diffraction pattern of *C. papyrus* fibers showing the characteristic crystallographic planes of cellulose I.

The X-ray diffraction (Figure 6) analysis of *C. papyrus* fibers reveals characteristic peaks of cellulose I, mainly in the regions associated with the (1−10)/(110) and (200) crystallographic planes, which are typical of plant fibers [54]. These peaks reflect the coexistence of well-organized crystalline regions and amorphous domains, which are characteristic of lignocellulosic fibers. The diffraction profile reflects the structural organization of cellulose microfibrils within the fiber cell wall and enables the evaluation of the crystalline fraction of the material. The crystallinity index calculated by the Segal method, although widely employed, may overestimate the crystalline fraction due to peak overlap and the influence of crystallite size [55,56]. Despite these limitations, the diffraction pattern indicates a relatively high degree of cellulose organization, which, according to the literature, is an important structural characteristic associated with the performance of lignocellulosic fibers in engineering applications [56,57].

The crystallinity index (CI) of cellulose in the fibers, calculated according to Equation (2) using the baseline-corrected diffractogram, was approximately 70.15%. Other authors have studied the crystallinity index using Segal’s method in other *Cyperus* species and found lower values. In the study by Mayandi et al. [53] on *C. pangorei* fibers, a crystallinity index of 41% was reported, comparable to the results of Bhunia et al. [58] for *C. platystylis* (41.12%) and Bhunia et al. [59] for *C. digitatus* and *C. exaltatus* (41.06% and 39.67%, respectively). The relatively higher crystallinity index observed for *C. papyrus* may be associated with a greater proportion of ordered cellulose domains relative to amorphous regions, reflecting differences in the supramolecular organization of cellulose microfibrils. Such differences may arise from variations in cellulose content, plant maturity, genetic background, environmental growing conditions, and fiber development. Furthermore, although the Segal method is widely used for comparative purposes, differences in peak overlap and crystallite size may also influence the calculated crystallinity index [56].

More broadly, the crystallinity index of papyrus fibers is higher than that reported for jute (57.53%) [60] and approaches the values observed for other fibers frequently used in composites, such as flax (85.1%) [61] and hemp (85.25%) [62]. Although crystallinity should not be considered in isolation, the degree of cellulose structural organization can influence the mechanical behavior and other physicochemical properties of cellulosic materials [56]. Although no direct statistical relationship between crystallinity and the mechanical properties was established in the present study, the relatively high crystallinity index indicates a well-organized cellulose structure and supports the potential of *C. papyrus* fibers as sustainable reinforcement for polymer composites.

### 3.7. FTIR

**Figure 7:**
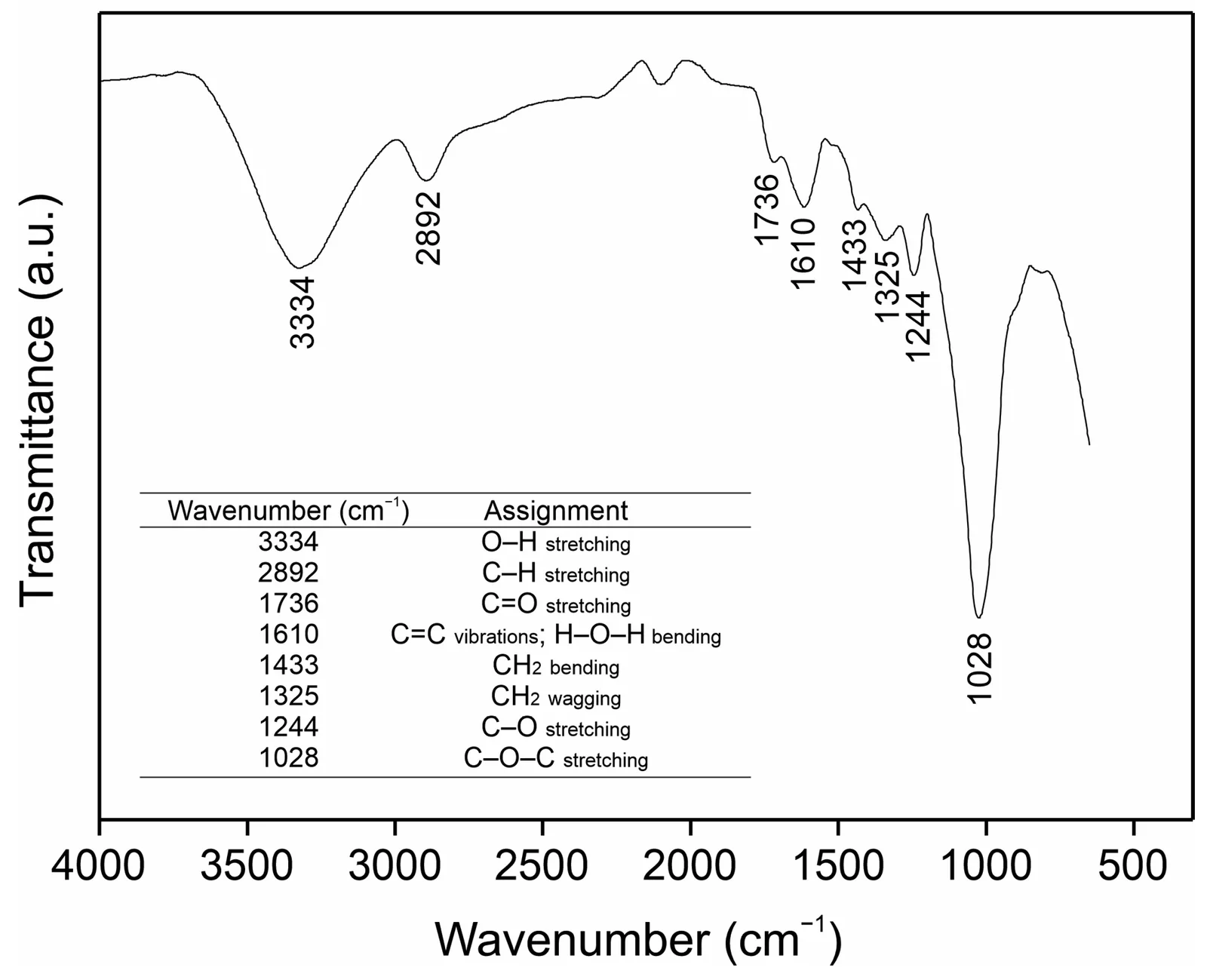
FTIR spectrum of *Cyperus papyrus* fibers and the corresponding assignments of the main absorption bands.

Figure 7 shows the FTIR spectrum obtained for *C. papyrus* fibers and the assignments of the principal absorption bands. Overall, the spectrum exhibits the characteristic profile of an untreated lignocellulosic material, reflecting the simultaneous presence of cellulose, hemicellulose, lignin, and minor extractive components. Similar spectral features have recently been reported for fibers extracted from other species of the genus *Cyperus* [58] and for different raw cellulosic fibers [63].

The broad and intense band centered at 3334 cm^−1^ is assigned to O–H stretching vibrations of hydroxyl groups present primarily in cellulose and hemicellulose, with possible contributions from phenolic hydroxyl groups in lignin and physically adsorbed water [58,64,65]. The broad profile of this band reflects the occurrence of intermolecular and intramolecular hydrogen bonding within the lignocellulosic structure [65]. These hydroxyl-rich constituents contribute to the hydrophilic character of natural fibers and may influence moisture absorption and interfacial interactions when the fibers are incorporated into polymeric matrices. The absorption band at 2892 cm^−1^ is associated with the symmetric and asymmetric stretching vibrations of aliphatic C–H bonds in methylene groups present in cellulose and hemicellulose [66]. The simultaneous occurrence of the bands at 3334 and 2892 cm^−1^ is therefore consistent with the carbohydrate-rich nature of the papyrus fibers and corroborates the predominance of α-cellulose observed in the chemical-composition analysis. These findings are consistent with the polysaccharide-rich composition expected for untreated lignocellulosic fibers. A weak feature was also observed near 2104 cm^−1^. Because this absorption is not commonly reported as a characteristic band of cellulose, hemicellulose, or lignin, and no complementary analysis was performed to determine its origin, no specific structural assignment was proposed.

The absorption band at 1736 cm^−1^ corresponds to the C=O stretching vibration attributed to acetyl groups in hemicellulose and carbonyl groups associated with lignin [67]. The band at 1610 cm^−1^ may result from overlapping contributions of aromatic skeletal C=C vibrations of lignin and H–O–H bending vibrations of water retained within the lignocellulosic structure [65,67]. Because natural fibers contain numerous accessible hydroxyl groups, residual or adsorbed water can contribute to absorption in this spectral region. Therefore, this band should not be attributed exclusively to lignin.

The C–H deformation bands corresponding to methyl, methylene, and methoxyl groups in lignin are observed at 1433 cm^−1^. The absorption feature near 1325 cm^−1^ is assigned to CH_2_ wagging vibrations associated with cellulose and hemicellulose, reflecting the polysaccharide framework of the lignocellulosic cell wall [68]. The band at approximately 1244 cm^−1^ corresponds to the C–O stretching vibration of the acetyl group present in both lignin and hemicellulose [44]. The peak at 1028 cm^−1^, previously reported by Machmudah et al. [69], is assigned to C–O and C–O–C stretching vibrations associated with glycosidic linkages in cellulose, hemicellulose, and lignocellulosic carbohydrate structures.

### 3.8. Comparative Performance of Papyrus and Recently Characterized Lignocellulosic Fibers

Table 3 compares the chemical, structural, physical, mechanical, and thermal properties of *Cyperus papyrus* with those of recently characterized lignocellulosic fibers investigated as reinforcement for polymer composites. Although direct comparisons among natural fibers should be interpreted with caution because their properties are influenced by plant maturity, environmental and growth conditions, fiber extraction procedures, and testing methodologies, this comparison provides a useful framework for positioning the performance of papyrus fibers relative to other lignocellulosic reinforcements.

Compared with the selected fibers, *C. papyrus* exhibited one of the highest α-cellulose contents and a crystallinity index comparable to that of Mariscus culms. The fiber density was intermediate among the compared materials, remaining lower than those of reddish shell bean, vetiver grasses, and palm leaf stalk fibers, but higher than those of spathe date palm and Mariscus culms. Likewise, the average fiber diameter was among the smallest values reported. Collectively, these characteristics indicate that papyrus fibers exhibit a combination of chemical composition, structural organization, and physical properties comparable to those of recently characterized lignocellulosic fibers.

These structural and physical characteristics are reflected in the mechanical performance of the fibers. Papyrus exhibited an average tensile strength (142 MPa), exceeding the values reported for spathe date palm, reddish shell bean, and Mariscus culms, while remaining below those of vetiver grasses and palm leaf stalk fibers. In contrast, Young’s modulus (11 GPa) was comparable to that of vetiver grasses and higher than those reported for the other fibers included in the comparison. These results indicate that *C. papyrus* combines moderate tensile strength with relatively high stiffness, demonstrating a balanced mechanical performance among recently characterized lignocellulosic fibers. Such behavior is particularly relevant for semi-structural composite applications, where stiffness and dimensional stability are often as important as tensile strength for maintaining structural integrity under service conditions [75].

In addition to mechanical performance, thermal stability is another important parameter for evaluating the suitability of natural fibers as reinforcement in polymer composites. Although papyrus exhibited the lowest thermal stability among the evaluated fibers, its degradation onset temperature (220 °C) remains compatible with the processing temperatures commonly employed for thermoplastic matrices such as polyethylene, polypropylene, and poly(lactic acid), whose processing temperatures generally remain below the onset of thermal degradation of lignocellulosic fibers [76].

Beyond the direct comparison of property values, some characteristics of *C. papyrus* should also be considered in relation to future composite processing. The significant dependence of tensile performance on fiber diameter indicates that dimensional variability may influence reinforcement selection and should be considered during fiber preparation. The relatively high α-cellulose content and crystallinity index reflect the structural organization of the fiber, although no direct statistical relationship between crystallinity and mechanical performance was established in the present study. In addition, the hydroxyl-rich chemical structure identified by FTIR indicates the hydrophilic character of the fibers, which may affect compatibility with hydrophobic polymer matrices and may require consideration of surface modification or matrix selection in future composite formulations.

## 4. Conclusions

The results demonstrate that *Cyperus papyrus* fibers exhibit structural, chemical, thermal, and mechanical characteristics that support their potential as sustainable reinforcements for polymeric composites. An inverse relationship was observed between fiber diameter and tensile strength, while thinner fiber classes also exhibited higher Young’s modulus values.

The average fiber diameter was 164.87 µm. The average tensile strength was 141.95 ± 89.33 MPa and the average Young’s modulus was 11.15 GPa, with thinner fibers exhibiting superior mechanical performance. The crystallinity index was 70.15%, while the fibers exhibited thermal stability up to approximately 220 °C. Although the tensile strength of *C. papyrus* fibers is lower than that reported for several natural fibers commonly used as composite reinforcements, the combination of satisfactory stiffness, relatively high crystallinity, suitable thermal stability, and renewable origin highlights their potential as a sustainable reinforcement for polymeric composites.

The characterization was limited to untreated *C. papyrus* fibers. Future studies should investigate their performance as reinforcement in polymer composites, including fiber–matrix interfacial adhesion, the effects of surface modification and chemical treatments, and the long-term durability of the resulting composites.

## Figures and Tables

**Table 1:** Diameter classes established according to the Sturges criterion and used for tensile characterization.

Class	Diameter Interval (µm)
C1	71.65–102.92
C2	>102.92–134.18
C3	>134.18–165.45
C4	>165.45–196.72
C5	>196.72–227.98
C6	>227.98–259.25
C7	>259.25–290.52
C8	>290.52–321.79

**Table 2:** Chemical characteristics and relative density of fibers from *Cyperus* species.

Component	*C. papyrus*	*C. papyrus*	*C. esculentus*	*C. dichrostachus*
	Present Work	[11]	[38]	[41]
α-Cellulose (%)	63.30 (±3.11)	61.82	47.20	60.27
Hemicellulose (%)	25.88 (±1.15)	11.82	13.00	22.72
Lignin (%)	15.44 (±0.04)	23.61	29.30	16.59
Extractives (%)	6.81 (±0.14)	2.75	3.90	1.55
Relative density (g/cm^3^)	1.12 (±0.10)	1.15	-	1.22

**Table 3:** Comparison of the main properties of *C. papyrus* and selected lignocellulosic fibers for potential use as polymer composite reinforcements.

Characteristic	Papyrus	Spathe Date Palm	Reddish Shell Bean	Culms of Mariscus	Vetiver Grasses	Palm Leaf Stalks
	Present Work	[70]	[71]	[72]	[73]	[74]
α-Cellulose (%)	63.3	*	*	58.3	42.7	63.7
Crystallinity index (%)	70.2	57.8	57.0	72.2	67.0	31.4
Density (kg·m^−3^)	1120	590	1580	769	1498	1473
Average diameter (µm)	165	151	786	244	984	381
Tensile strength (MPa)	142	100	111	134	189	249
Young’s modulus (GPa)	11	4	6	5	10	*
Thermal stability (°C)	220	270	328	258	244	326

* Indicates that the property was not reported in the original study.

## Data Availability

The original contributions presented in this study are included in the article. Further inquiries can be directed to the corresponding author.

## Abbreviations

The following abbreviations are used in this manuscript:

CI	Crystallinity Index
TGA	Thermogravimetric Analysis
DTG	Derivative Thermogravimetry
SEM	Scanning Electron Microscopy
FTIR	Fourier Transform Infrared Spectroscopy
XRD	X-ray Diffraction
